# Regularized Least Squares Cancer Classifiers from DNA microarray data

**DOI:** 10.1186/1471-2105-6-S4-S2

**Published:** 2005-12-01

**Authors:** Nicola Ancona, Rosalia Maglietta, Annarita D'Addabbo, Sabino Liuni, Graziano Pesole

**Affiliations:** 1 Istituto di Studi sui Sistemi Intelligenti per I'Automazione, CNR, Via Amendola 122/D-I, 70126 Bari, Italy; 2 Istituto di Tecnologie Biomediche-Sezione di Bari, CNR, Via Amendola 122/D, 70126 Bari Italy; 3 Dipartimento Scienze Biomolecolari e Biotecnologie, Universitá di Milano, Via Caloria 26, 20133 Milano, Italy

## Abstract

**Background:**

The advent of the technology of DNA microarrays constitutes an epochal change in the classification and discovery of different types of cancer because the information provided by DNA microarrays allows an approach to the problem of cancer analysis from a quantitative rather than qualitative point of view. Cancer classification requires well founded mathematical methods which are able to predict the status of new specimens with high significance levels starting from a limited number of data. In this paper we assess the performances of Regularized Least Squares (RLS) classifiers, originally proposed in regularization theory, by comparing them with Support Vector Machines (SVM), the state-of-the-art supervised learning technique for cancer classification by DNA microarray data. The performances of both approaches have been also investigated with respect to the number of selected genes and different gene selection strategies.

**Results:**

We show that RLS classifiers have performances comparable to those of SVM classifiers as the Leave-One-Out (LOO) error evaluated on three different data sets shows. The main advantage of RLS machines is that for solving a classification problem they use a linear system of order equal to either the number of features or the number of training examples. Moreover, RLS machines allow to get an exact measure of the LOO error with just one training.

**Conclusion:**

RLS classifiers are a valuable alternative to SVM classifiers for the problem of cancer classification by gene expression data, due to their simplicity and low computational complexity. Moreover, RLS classifiers show generalization ability comparable to the ones of SVM classifiers also in the case the classification of new specimens involves very few gene expression levels.

## Background

The advent of the technology of DNA microarrays constitutes an epochal change in the study, treatment, analysis, classification and discovery of different types of cancer. It is well understood that cancer classification is a crucial step for cancer diagnosis and treatment [1,2]. Conventional classification of cancer has been based primarily on examination of the morphological appearance of tissue specimens, but this method suffers of serious limitations. It is subjective and depends on highly trained pathologists. Moreover, tumors with similar histopathological appearances can follow different clinical courses and show different responses to therapy [3]. The information provided by DNA microarrays allows to approach the problem of cancer diagnosis and treatment from a *quantitative *rather than *qualitative *point of view. The importance of the information embedded in gene expression data provided by DNA microarrays for identifying new cancer classes and for automatically classifying tumors to known classes was firstly pointed out by Golub in [1]. In tumor classification, the problem is to assign a label *y*, for example normal or cancerous tissue, to a new gene expression pattern **x**, starting from the knowledge of ℓ *examples S *= {(**x**_1_, *y*_1_), (**x**_2_, *y*_2_),...(**x**_ℓ_, *y*_ℓ_)} whose association between the gene expression pattern **x**_*i *_and its relative class label *y*_*i *_is known in advance. Here **x **is a vector whose components indicate the gene expression levels provided by a DNA microarray. Under this perspective, the problem of cancer classification can be seen as a supervised learning problem, or a *learning from examples *problem [4], in which the goal is to determine a separating surface, optimal under certain conditions, which is able to discriminate normal from cancer tissues, or to distinguish among different types of tumors. In this paper we focus only on two class classification problems, since multi-class problems can be seen as a straightforward generalization of two-class problems. Before introducing the main aspects of our work, it is worth to point out that the ultimate goal of any classifier, and in general of any learning machine, is *to generalize*, that is to predict the correct output *y *relative to never seen before input patterns **x**, by using a training set *S *composed of a *finite *number of examples. Thus the central problem is not classifying the training data in *S*, because any sufficiently complex learning machine could separate *S *without errors. The crucial problem is to design classifiers having low error rate on new data. In the context of classification of DNA microarrays, such a problem is even more challenging because typically the number of examples is relatively small and the dimensionality, i.e. the number of genes whose expression levels are measured, is very large.

Statistical learning theory [5] provides a valuable non asymptotic theory for asking questions about the accuracy of models built when a limited amount of data is available. In this general framework, Support Vector Machine (SVM) classifiers provide excellent performances in terms of generalization error in different application domains such as object detection in images [6,7], odor classification [8], pedestrian detection [9], etc. In particular, in the context of cancer classification from gene expression data it outperforms many well known approaches [10-13] and it has to be considered as the method of reference for evaluating new techniques. The basic idea of statistical learning theory is very simple: for a finite set of training examples, the search for the best model or approximating function has to be constrained by an appropriately small hypothesis space, that is the set of functions the machine implements. If the space is too large, functions can be found which fit exactly the data, but they will have poor generalization capabilities on new data. SVM implements such an idea determining the classifier minimizing both the error on the training set (empirical risk) and the complexity of the hypothesis space.

Another approach to classification and in general to the problem of approximating a multivariate function from sparse data and in the presence of noise is regularization theory [14-16]. Also in this framework we need to constraint the hypothesis space for finding a suitable approximating function from a finite number of training examples. Such a constraint takes the form of a smoothness functional measuring the complexity of functions belonging to the chosen hypothesis space. In this general framework, Regularized Least-Squares (RLS) classifiers [17] provide a highly viable alternative to SVMs because they enjoy a number of suitable properties such as simplicity and reliability.

A first comparison between SVM and RLS classifiers can be found in [18]. In their analysis, the authors used very simple bench-mark data sets having characteristics very different from the ones relative to the cancer classification problem by gene expression data. In fact, they used data sets having a ratio between number of examples and number of components ranging from 3.5 for the sonar data set to 96 for the pima indian data set. Such ratios are very far from the ratio of order of 1/100 that is typical for the problem we are considering here. So from their study we can not infer any consequence about the performances of the RLS classifiers on the problem at hand. In this paper we compare SVM and RLS classifiers for the specific problem of cancer classification by gene expression data. In the context of supervised learning models, as the ones we are considering here, particularly important is the quantity to measure for comparing two machines. We know that two machines have similar performances if their generalization errors are comparable. As we will show in the next sections [5], a measure of the generalization error of any supervised learning machine is the *risk *and so models showing the same risk have comparable performances. However, the risk functional, as usually defined, has not a practical usefulness because it involves the knowledge of the probability distribution function underlying the data that is in general unknown. Nevertheless, we can adopt the Leave-One-Out (LOO) procedure which uses the available data for evaluating the generalization error of a machine. In fact, as the Luntz and Brailovsky theorem shows [19], the LOO error is an almost unbiased estimator of the risk and so it is a practical procedure for assessing the performances of a supervised learning machine from a finite number of data. Based on this estimator we show that RLS and SVM models have similar generalization abilities. The comparison involved three different data sets described in [1,12,20]. The experimental results suggest that we can benefit of the simplicity of RLS machines maintaining the same prediction error of SVM. The main advantage of RLS machines is that for solving a classification problem we need to solve a single linear system of order equal to either the number of features or the number of training examples. This is in contrast to SVM approach which requires the solution of a quadratic programming problem with linear constraints. Moreover and more important, RLS machines allow to get an exact measure of the leave-one-out error with just one training. In the case of SVM, such important measure requires the training of a number of machines equal to the number of training examples. At the aim of fully assessing both the classification models, we analyze their performances with respect to the number of genes, selected with different gene selection strategies. Note that the focus here is not on the determination of the optimal number of genes for classifying tissues belonging to a given tumor class. For this reason others and more sophisticated methodologies have to be adopted which take into account the bias selection problem [21]. Here we want to show that both models have comparable performances even when a very few number of genes is used for classifying. Following the statistical approach outlined by Golub and its co-workers in [1,2,12], we adopt non-parametric permutation tests for studying how many and what genes have to be used for classifying. The problem of identifying the gene signature concerning a particular type of cancer is out of the scope of the present work.

## Methods

### Classification models

Due to the particular problem of cancer classification from gene expression data in which we have a small number of training examples, each one with very large dimensionality, we limit our attention to linear classifiers. Nevertheless the methods we are going to illustrate can be easily generalized for designing non-linear classifiers.

We are given a training set  of size ℓ where **x**_*i *_∊ ℝ^*n *^and *y*_*i *_∊ {-1,1}, for *i *= 1,2,...,ℓ. In the simplest case of linearly separable set *S*, the classification problem consists of determining a hyperplane **w**·**x **+ *b *= 0, where **w **∊ ℝ^*n *^and *b *∊ ℝ, such that: *y_i _*= sign(**w**·**x**_*i *_+ *b*) for *i *= 1,2,...,ℓ, where sign (*x*) is 1 if *x *≥ 0 and -1 otherwise. Actually, classification is an ill-posed problem [14] because an infinite number of solution exist and then some constraint has to be imposed to the problem for making the solution unique.

#### SVM classification

The constraint imposed by SVM on the classification problem is the following: the solution has to maximize the distances with the closest points of *S*. The optimal separating hyperplane found by SVM, in the case of linearly separable set *S*, is the one maximizing the *margin m*, where *m = *2/||**w**|| is the distance between the hyperplane and the closest points of *S*. In the general hypothesis of non linearly separable classes, the optimal separating hyperplane w*·**x **+ b* = 0 found by SVM is solution of the following quadratic programming (QP) problem P1 with linear constraints:



where ***D ***is a matrix of size ℓ × ℓ, with *D*_*ij *_= *y*_*i*_*y*_*j*_**x**_*i*_·**x**_*j *_for *i,j *= 1, 2, ...,ℓ and ***λ ***= (*λ*_1_, *λ*_2_,..., *λ*_ℓ_)^⊤ ^is a vector of ℓ non negative Lagrange multipliers. The regularization parameter *C *is the only free parameter and its value can be chosen by using cross validation. Let λ* be the solution of the considered problem P1. So the optimal w* is:



and the optimal b* is given by:

b* = *y*_*i *_- w*·**x**_*i *_    (2)

for each *i *such that 0 < <*C*. The points **x**_*i *_with  > 0 live on the margin of the classes and they are called *support vectors*. The classification of a new data **x **involves the evaluation of the decision function:

*y *= sign(*f*(**x**))     (3)

where:



#### RLS classification

RLS models [14] were proposed mainly for facing regression problems. The main difference between a regression and classification problem is that in the former the output variable *y *can assume any real value; in the latter, it can assume a finite number of possible values. In our case, *y *assumes only two values {-1,1}. This means that every classification problem can be considered as a regression problem. In the case of linear regression, we want to determine the function *f*(**x**) = **w**·**x**, with **w **∊ ℝ^*n*^, which approximates the examples in *S *in the least squares sense. This is equivalent to solving the following constrained minimization problem P2:



*subject to *||**w**||^2 ^≤ *α*

where *α *∊ ℝ and ||**w**|| =  is the Euclidean norm induced by the scalar product ·. Note that the bias term is implicitly present in our model by including a component constant and equal to one to the input vectors. Before solving the problem P2, some considerations are in order. The objective function that we minimize, in this particular case, takes the form of the mean square error of the predictor *y *= **w**·**x **evaluated on the training data. Here the error is expressed as the square *deviation*, *ε*_*i *_= (*y*_*i *_- **w**·**x**_*i*_)^2^, between the target value *y_*i *_*and the value of the predictor **w**·**x_*i*_**. Let *d_i _*be the square *distance *between the generic input data **x**_*i *_and the approximating hyperplane *y *= **w**·**x**, where by definition:



This equation shows that the smaller ||**w**||^2^, the better the deviation *ε*_*i *_approximates the true distance *d*_*i*_. This is the reason why we introduce the constraint ||**w**||^2 ^≤ *α*. In this way the optimal approximating hyperplane solution of the constrained problem is the hyperplane minimizing the mean square distance with the training points. For determining **w **∊ ℝ^n ^solution of P2, let us consider the Lagrangian function:



The vector **w **minimizing (5) is solution of the following linear system of order *n*:

(**XX**^⊤ ^+ *λ*ℓ**I**_*n*_) **w **= **Xy **    (6)

where **X **is a *n *× ℓ matrix having the examples **x**_*i *_as its columns, **y **= (*y*_1_, *y*_2 _,...,*y*_ℓ_)^⊤ ^and **I**_*n *_is the *n *× *n *identity matrix. Note that, since the matrix **XX**^⊤ ^is positive semidefinite, then for *λ *> 0 the matrix **XX**^⊤ ^+ *λ*ℓ**I**_*n *_is definite positive and therefore invertible. Then the vector w* solution of the problem P2 exists and it is given by:

w* = (**XX**^⊤ ^+ *λ*ℓ**I**_*n*_)^-1^**Xy **    (7)

It is possible to show that the value of *λ *controls the influence of the noise present in the data on the estimation of the solution w*. The parameter *λ*, called regularization parameter, is the only free parameter and its value can be chosen by using cross validation. Analogously to SVM, the classification of a new data **x **involves the evaluation of the decision function:

*y *= sign(w*·**x**)     (8)

As equation (7) shows, determining w* requires the solution of a linear system of *n *order, where *n *is the number of components of each **x**_*i*_. In some cases *n *could be extremely large and so any direct method can be adopted for estimating w*. This occurs in the problem at hand where the number of genes *n *of each specimen is order of tens of thousand and the number ℓ of specimens is order of ten or hundred. We will show that the models we are describing allow to rewrite a linear system of *n *order as a linear system of ℓ order, overcoming the difficulties connected to problems with a huge number of features. At this aim, let us suppose **w **to be expressed as linear combination of the vectors **x**_*i *_for *i *= 1,2, ...,ℓ. This means that there exist ℓ coefficients **c **= (*c*_1_, *c*_2_,...,*c*_ℓ_)^⊤ ^such that:

**w **= **Xc **    (9)

Substituting (9) in (6) we have:

(**K **+ *λ*ℓ**I**_ℓ_) **c **= **y **    (10)

where **K **= **X**^⊤^**X **is a ℓ × ℓ matrix with generic element **K**_*ij *_= **x**_*i*_·**x**_*j *_and **I**_ℓ _is the identity matrix of ℓ order. Also in this case, since **K **is a positive semidefinite matrix, then for *λ *> 0 the matrix **K **+ *λ*ℓ**I**_ℓ _is positive definite and so invertible. Then the vector c* ∊ ℝ^l ^solution of (10) is given by:

c* = (**K **+ *λ*ℓ**I**_ℓ_)^-1^**y **    (11)

obtained by solving a linear system of ℓ order. Note that the normal w* to the optimal approximating hyperplane can be recovered by using (9). In this case the classification of a new data **x **involves the evaluation of the decision function:



#### Comparison between SVM and RLS classifiers

Numerous differences exist between these two classification models, but we will only mention some of these which are relevant for our discussion. The first difference consists in the method employed for determining the optimal **w**. SVM requires the solution of a QP problem with linear constraints of order ℓ, while RLS requires the solution of a system of linear equation of order ℓ or *n*. In the former, the complexity in solving problem P1 is independent of *n*. Moreover, when the number ℓ of examples is extremely large, decomposition methods can be applied for determining the exact solution [22]. In the latter, the complexity depends on ℓ and *n*. When both these quantities assume large values, iterative schemes have to be adopted for solving the system (6) or (10), so providing only approximated solutions.

The second difference consists in the representation of the optimal **w**. In SVM (see equation (1)), the solution is *sparse *meaning that it is expressed as linear combination of a fraction of the training examples (support vectors). In RLS (see equation (9)), on the contrary, the solution is *dense *meaning that it is expressed as linear combination of all training examples.

### Leave-one-out error

As we have already said in the introduction, the ultimate goal of a supervised learning machine *y *= *f *(**x**, ***α***) is *to generalize*, that is to correctly predict the output *y *corresponding to never seen before input patterns **x**. Here ***α ***is a parameter vector which the machine depends on, for example *C *in SVM and *λ *in RLS classifiers. Then a comparison between different classification models has to involve the comparison of their generalization errors. A measure of the generalization error of such a machine *f *is the *risk R*[*f*] defined as the expected value of the loss function *V*(*y*,*f*(**x**, ***α***)) (see [5]):

*R*[*f*] = ∫*V*(*y*, *f*(**x**, ***α***))*p*(**x**, *y*)*d***x***dy *    (13)

where *p*(**x**, *y*) is the probability density function underlying the data. The particular form of the loss function depends on the problem at hand. In classification problems, the loss takes the form of:



In general the probability density function *p*(**x**, *y*) is unknown and so we are not able to evaluate the risk. The only data we have are ℓ observations (examples)  of the random variables **x **and *y *drawn according to *p*(**x**, *y*). The leave-one-out (LOO) error provides a measure of the generalization error of a learning machine by using the ℓ observations in *S*. In fact, as the Luntz and Brailovsky theorem shows [19], the LOO error is an almost unbiased estimator of the risk (13) and it allows of assessing the performances of a supervised learning machine from a finite number of data. The computation of the LOO error is very simple. For every *i *= 1, 2, ..., ℓ, let  be the machine trained on the set *S*^*i *^= {(**x**_1_, *y*_1_),...,(**x**_*i*-1_, *y*_*i*-1_), (**x**_*i*+1_, *y*_*i*+1_),...,(**x**_ℓ_, *y*_ℓ_)} obtained from *S *removing the *i*-th example. Test the function  on the left out example (**x**_*i*_, *y*_*i*_) and measure the value of the loss function *V*(*y*_*i*_,  (**x**_*i*_, ***α***)). Repeat this procedure for each of the ℓ examples of the training set and sum the errors made. This number is the LOO error:



Note that  is the quantity that we have to compute for measuring the performances of any supervised learning machine, because it provides an estimate of the risk or generalization error associated to the selected machine. Moreover, this is the procedure of choice for estimating the unknown parameter vector ***α ***which the machine depends on. In fact, for a fixed training set, the generalization error of the machine is a function of ***α***. Then, the best parameter vector α* will be the one minimizing .

#### LOO-error for RLS classifiers

Although the LOO error enjoys several interesting properties, its computation is tremendously expensive because it requires of training a number of machines equal to the number of training examples. In the case of RLS classifiers, the LOO error can be calculated in an exact way just training a single machine by using all the training examples. In fact it can be showed [15] that:



where *f_s _*is the machine trained on *S *and **G **= (**K **+ *λ*ℓ**I**_ℓ_)^-1^. This is a fundamental property of the RLS classifiers because it allows to evaluate the generalization ability of a classifier without any additional cost.

### Gene selection

A very important question in cancer classification problem is determining which genes are the most relevant in identifying a specimen or a particular disease. This is an open problem, relevant for several reasons both biological and computational. Finding genes which expression levels correlate with a particular disease is important for understanding the disease and for choosing the most appropriate treatment. Furthermore, classifying a specimen on the basis on few expression levels could in principle improve the performances of the classifier, eliminating the noise associated to irrelevant genes. Gene selection is a particular instance of a more general problem known in machine learning as *feature selection*. In general, the methods for selecting features can be grouped in two main categories: filter methods and wrapper methods [23]. Filter methods select features by using criteria independent of the ones used in the classification stage. Wrapper methods, on the contrary, use the same or similar criteria as the ones used by the classifier. In this paper we focus on two different feature selection approaches. The first one [1,2], known as *signal-to-noise *(S2N), is a filter method and it is based on the following statistic:



where *j *is the gene index. (*μ*_+1_(*j*),*σ*_+1_(*j*)) and *(μ*_-1_(*j*),*σ*_-1_(*j*)) are the mean and the standard deviation of the expression levels of the *j*-th gene in the positive and negative examples respectively. Genes *x*_*j *_highly correlated with the class label or more relevant for classifying are expected to provide large values of |*T*_*S*2*N*_(*j*)| The second approach we consider is a variant of *recursive feature elimination *(RFE) strategy proposed in [24]. It is a wrapper method and it is based on the following statistic:

*T*_*w*_(*j*) = *w*_*j *_   *j *= l,2,...,*n *    (18)

where **w **is the normal of the optimal separating hyperplane found by SVM or RLS methods. The idea underlying this approach is very simple. We know that the label *y *associated to a new input **x **is given by . So, if the gene expression levels have similar ranges, genes having large values of |*w*_*j*_| are more important than others in determining the class label. Instead of using a recursive approach for selecting the most relevant genes as suggested in [24], we use a more greedy strategy consisting in training the machine one time only by using all the available genes and selecting the most informative features according to the obtained **w**. For this reason we call our approach *not-RFE *(NRFE). In both strategies, the genes are ranked in decreasing order according to the selected statistic and the highest values correspond to the most relevant genes.

### Number of relevant genes

So far we have described two statistics for ranking genes based on their expression levels in both classes. Now, in order to determine how many genes are really important for classifying a given specimen, we apply a common method in classical statistics named hypothesis testing (see [1]). The idea is to hypothesize that there is no dependency between expression levels and class labels, and to consider relevant for the classification those genes which reject such hypothesis. At this aim, we define the null hypothesis *H*_0 _in which we assume that the random variables *x *and *y *are independent or equivalently that the class conditional probability density functions are identical. The goal of hypothesis test is to reject *H*_0 _at a given level of significance *α*, where *α *is the probability of rejecting the null hypothesis when it is true, that is of declaring that the *x *and *y *are uncorrelated when they are not. Let *t*_0 _be the observed value of the statistic *T *as computed on the data set *S*, *t*_0 _= *T*(*x*_1_, *y*_1_, *x*_2_, *y*_2_,...,*x*_ℓ_, *y*_ℓ_), and let *p*_0 _= *P*_*T*_(*T *≥ *t*_0_) be the corresponding *p-value*, that is the probability that *T *is grater than or equal to *t*_0_. Note that *P*_*T *_is the distribution function of the random variable *T *under the null hypothesis. If *p*_0 _≤ *α *then we reject *H*_0 _at level of significance *α*.

The application of the hypothesis testing method requires the knowledge of the density or distribution function of the adopted *T *statistic under the null hypothesis. When the density of the adopted statistic is unknown or when the data do not verify the hypotheses which the statistic is based on, then we have to the invoke nonparametric permutation tests [25]. This nonparametric technique allows to estimate the probability density function of any statistic, under the null hypothesis, from the available data. The reason which justifies this procedure for estimating the density *p*_*T*_(*t*) is that under the null hypothesis, since the random variables *x *and *y *are independent, all the training set generated through permutations are equally likely to be observed.

## Results

### Data sets description

The above mentioned classification techniques have been applied to different cancer diagnosis problems. Three benchmark data sets have been considered. The first one, named 'Leukemia data set' [1], concerns the classification of acute leukemias into acute myeloid leukemia (AML) and acute lymphoblastic one (ALL). It consists of 38 bone marrow samples (11 AML, 27 ALL) obtained from acute leukemia patients at the time of diagnosis (i.e. before that any treatment was used). These samples are used as training set. An additional set, composed of 14 AML and 20 ALL samples, is utilized to test the classifiers. Each sample is a vector composed by 7129 elements, each one corresponding to the log_10 _normalized expression value of a gene. This data set has been extensively analyzed in literature [2] also by using machine learning techniques [10]. Much more details about this data set and a complete breakdown of microarray composition can be found on the web site . The second data, named 'Colon data set' [20], regards the problem of classifying tumor and normal colon tissues. It is composed by 40 tumor and 22 normal colon tissue samples. Each sample consists of 2000 human gene expression levels. The data set and more detailed information on it are available on the web at site . The last analyzed data set is relative to the classification of different malignancies samples against normal tissue ones [12], it will be identified as 'Multi-cancer data set'. It is composed by 280 samples: 190 examples are relative to cancer tissues, spanning 14 common tumor types, the remaining 90 samples represent normal tissues. Each example in this data set consists of the expression levels of 16063 genes. Complete details regarding patient samples, pathology, molecular biology protocols, data analysis and additional information are available at site . It is worth nothing that, in the present work, this data set is analyzed in order to perform a two-class classification problem (i.e. to discriminate between diseased and normal samples).

### Results on the Leukemia data set

First of all, we used all of 7129 gene expression levels present in each specimen. We trained SVM classifiers on the 38 samples in the training set for different values of *C *parameter, measuring for each one the empirical risk and the LOO error given by equation (15). The training set is linearly separable and the LOO error reaches its minimum value of 1 (see table 1) in correspondence of *C *= 1e - 6. Then the best SVM classifier on this training set is the one obtained with *C *= 1e - 6 because it is the machine minimizing the LOO error. We tested such machine on the 34 points in the test set obtaining 1 error (see table 1). The same results are reported in [10], where the authors also noted that using SVM with polynomial kernel functions did not improve the performances.

The same procedure was carried out by using RLS machines. We trained RLS classifiers on the training set for different values of *λ *parameter and for each one we measured the empirical risk and the LOO error by using equation (16). The training set is linearly separable for each *λ *in the considered range. Moreover, the LOO error reaches its minimum value of 1 (see table 1) in correspondence of *λ *= 1. Then the best RLS classifier on this training set is the one obtained with *λ *= 1. We tested such machine on the test set obtaining 1 error as reported in table 1.

**Figure 1:**
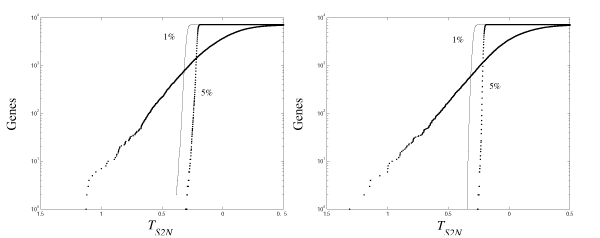
: Observed *T*_*s*2*N*_(*j*) distribution computed on the Leukemia data set, compared to randomly permuted class distinctions. The number of genes highly expressed in a) ALL and b) AML is shown on y-axis.

Successively, in order to carry out a most accurate analysis and to have a most complete insight about the performances of SVM and RLS machines on this data set, we have computed the LOO errors on the whole data set obtained putting together training and test examples. The values of the free parameters, corresponding to the best machines, are *C *= 45 and *λ *= 10 respectively. The LOO errors obtained by the best machines are reported in table 1 where clearly results that SVM and RLS behave exactly the same. In order to understand the influence of irrelevant genes on the performances of the classifiers, we considered some subsets of features. We established the number of genes to select applying permutation tests to the data, by using *T*_*S*2*N *_and *T*_*w *_statistics. Figure 1 depicts the values of *T*_*S*2*N *_statistic as computed on the actual data set and on randomly permuted class labels. The number of permutations of the labels was 1500. Genes more highly expressed in ALL are shown in the left picture, and those more highly expressed in AML are shown in the right picture. The large number of genes highly correlated with the class distinction is clear from the picture. Moreover, in both pictures, the curve of the observed statistic intersects the 5% curve about at 1000 genes, indicating that in the data set there are 1000 genes which reject *H*_0 _at significance level of *α *= 5%. Then we ranked the genes according to the absolute value of *T*_*S*2*N*_and chose the top *k *genes, with *k *equal to 1000, 100, 50, 40, 30, 20, 10, 5 and 3 genes. A similar analysis has been effectuated by using *T*_*w *_statistic. Here **w **was the parameter vector corresponding to the best RLS classifier, that is the one minimizing the LOO error on the current data set^1^. As picture 2 shows, *T*_*w *_is unable to disclose the correlation existing between gene expression level and class label, as *T*_*S*2*N *_does. Nevertheless, we equally measured the performances of SVM and RLS classifiers on genes selected by *T*_*w *_statistic. In fact, as noted in [2], in some particular cases, some genes may be truly predictive of the class label despite the lack of statistical significance in permutation tests. At the aim of testing this experimental evidence, we ranked the genes according to the absolute value of *T*_*w *_and chose the top *k *genes, with *k *equal to 1000, 100, 50, 40, 30, 20, 10, 5 and 3 genes. In table 2 we report the performances of SVM and RLS classifiers obtained on the Leukemia data set, for various number of genes selected by S2N and NRFE methods.

### Results on the Colon data set

First of all note that in this case, as in the following data set, we do not have the distinction between training and test set, because we have a single data set. For this reason, we do not report the test error but the LOO error only. We have primarily evaluated the performances of SVM and RLS classifiers on the Colon data set by using all the gene expression levels present in each specimen, successively we have consider opportune subsets of genes. The experimental results on the whole and reduced data sets are summarized in table 3.

**Figure 3:**
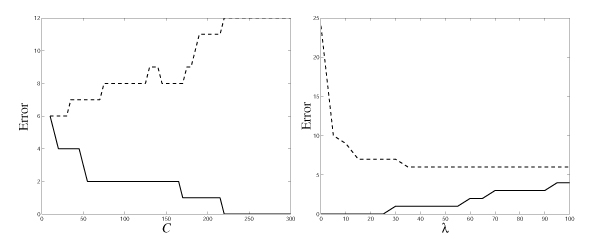
: LOO error (dotted line) and empirical risk (solid line) w.r.t the regularization parameter obtained on Colon data set by using a) SVM and b) RLS classifiers.

The behavior of the empirical risk and of the LOO error of SVM and RLS classifiers evaluated for different values of the regularization parameter are depicted in figure 3 for the whole Colon data set. Note that the data set is linearly separable. These plots give also a precious hint to fully understand the role of free parameters (*C *in SVM and *λ *in the RLS machines) by observing the empirical risk curves. In fact, increasing *C *in SVM the empirical risk decreases, whereas increasing *λ *in RLS the empirical risk increases. These behaviors of the empirical risk curves can be fully justified reminding that, in SVM, the *C *parameter can be thought of as the cost the machine pays for each training error. On the contrary, in the RLS machines, the same role is played by  (see equation 5).

In order to determine the number of relevant genes to be considered in the feature selection process, we have computed the *T*_*S*2*N *_statistic on the actual data set and in the hypothesis that *H*_0 _holds true. The number of label permutations was 2000. The observed statistic intersects the 5% curve in correspondence of 500. So 500 is the maximum number of genes which reject the null hypothesis at significance level of 5%. Then we ranked the genes according to the absolute value of *T*_*S*2*N *_and chose the top *k *genes, with *k *equal to 500, 400, 300, 200, 100, 50, 10 and 5 genes. A similar analysis has been effectuated by using *T*_*w *_statistic. Also in this case, this statistic shows poor capacity of revealing the existing correlation in the data. As in the previous analysis, we ranked the genes according to the absolute value of *T*_*w *_and chose the top *k *genes, with *k *equal to 500, 400, 300, 200, 100, 50, 10 and 5 genes.

### Results on the Multi-cancer data set

Primarily, the 16063 gene expression levels of each specimen have been used for classifying. The experimental results are summarized in table 4.

The data set is linearly separable. The best SVM corresponds to *C *= 3. The best RLS classifier corresponds to *λ *= 20.

It is important to note that the errors obtained on this data set are much greater than the one achieved in the data sets previously analyzed, probably reflecting the large complexity of the data due to the great degree of biological variability in gene expressions.

The non parametric permutation test was carried on the Multi-cancer data set, performing 1000 random permutations of the class labels. The maximum number of genes which rejects the null hypothesis at significance level of 5% is 1400. Then we ranked the genes according to the absolute value of *T*_*S*2*N *_and chose the top *k *genes, with *k *equal to 1400, 1000, 500, 300, 200, 100, 50 and 10 genes. The same numbers of genes were selected by using the *T*_*w *_statistic. The results on the reduced data sets are reported in table 4.

## Discussion

Some conclusions on the two classification algorithms can be drawn. The first and more important one is that, when the whole data sets are considered, both machines provide generalization errors comparable as the tables 1, 3 and 4 show. This indicates that RLS approach is able to determine classifiers with good generalization ability even in the case of very small training set, with a huge number of features.

Concerning the computational time, both techniques require a few seconds for determining the optimal classifier because, in the present context, the training involves only a few examples. The second consideration concerns the role of *λ *in RLS machines. This parameter de-facto controls the generalization ability of the RLS classifiers, exactly as *C *does in SVM ones. The figure 3 depicting the behavior of the LOO error shows this fact. We have observed similar behaviors of this quantity in all the experiments carried out which do not show for lack of space. Moreover, our analysis shows that standard least-square machines, obtained setting *λ *= 0, have very poor generalization abilities. In fact for *λ *= 0 all the considered RLS classifiers separate correctly the training data, but they show a very large LOO error. The main problem in machine learning is not to correctly classify the training data. The main problem is to generalize and RLS classifiers guarantee high generalization ability for appropriate values of the regularization parameter *λ*.

The performances of SVM and RLS classifiers continue to be comparable even though the number of genes used for classifying a specimen is extremely reduced. Tables 2, 3 and 4 confirm such a result. Moreover, as noted in all three data set, also a statistic which is not able to reveal statistically significant differences in the data can however select genes which increase the performances of the classifier. This is not surprising. The fact that a gene is relevant for classifying a given specimen does not involve the statistic, it involves the classification process. So, a gene is relevant for a classifier if its usage reduces the *generalization error *of the classifier, as measured by the LOO error. Any gene selection strategy has to guarantee that the subset of genes selected is the most appropriate for the chosen classifier, that is it is the subset of features minimizing the LOO error of the classifier. In this sense feature selection and parameter selection are two instances of the same problem which has as ultimate objective the one of reducing the generalization error of any learning machine.

## Conclusion

In this paper we have shown that RLS classifiers have performances comparable to the ones of SVM classifiers for the problem of cancer classification by gene expression data. The comparison has been carried on measuring the Leave-One-Out errors relative to each classifier obtained on three different real data set. The classification performance analysis involved the whole set of genes as well as suitable subsets of genes selected by different gene selection strategies. Our analysis suggests that RLS classifiers are a valuable alternative to SVM classifiers for the problem at hand due to their simplicity and low computational cost. Moreover, RLS classifiers show generalization errors comparable to the ones of SVM classifiers also in the case the classification of new specimens involve very few gene expression levels.

## List of abbreviations used

SVM: Support Vector Machines. RLS: Regularized Least Square. LOO: Leave One Out.

## Note

^1^Note that the *T*_*w *_statistic can be considered a filter method when the features it selects are input to SVM classifiers, and a wrapper method when the features are input to RLS classifiers.

## Figures and Tables

**Figure 2:**
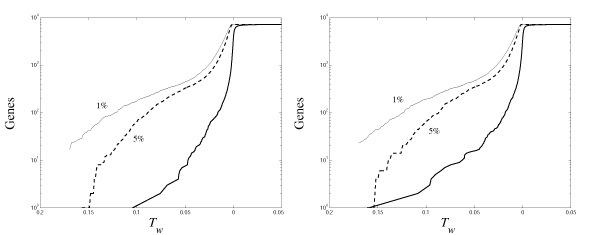
: Observed *Tw*(*j*) distribution computed on the Leukemia data set, compared to randomly per-mutated class distinctions. The number of genes highly expressed in a) ALL and b) AML is shown on y-axis.

**Table 1:** Minimum LOO error on the Leukemia training set (composed of 38 examples), error on the test set (34 examples) and minimum LOO error on the whole Leukemia data set (72 examples).

	SVM	RLS
LOO error on training set	1	1
Test error	1	1
LOO error on the whole data set	1	1

**Table 2:** Minimum LOO error computed on Leukemia data set (composed of 72 examples), for various number of genes, selected with S2N and NRFE statistics.

	SVM		RLS	
genes	S2N	NRFE	S2N	NRFE

1000	1	1	2	1
100	1	0	1	0
50	1	0	2	0
40	2	0	2	0
30	2	0	2	0
20	2	0	2	0
10	2	1	2	0
5	1	1	2	2
3	4	3	4	2

**Table 3:** Minimum LOO error computed on Colon data set (composed of 62 examples), for various number of genes, selected with S2N and NRFE statistics.

	SVM		RLS	
genes	S2N	NRFE	S2N	NRFE

2000	6		6	
500	6	6	7	6
400	7	6	6	6
300	6	6	6	6
200	7	6	7	4
100	9	5	7	4
50	8	4	6	1
10	6	6	7	5
5	7	8	7	8

**Table 4:** Minimum LOO error computed on Multi-cancer data set (composed of 280 examples), for various number of genes, selected with S2N and NRFE statistics.

	SVM		RLS	
genes	S2N	NRFE	S2N	NRFE

16063	105		90	
1400	46	40	59	49
1000	42	41	57	50
500	50	41	57	56
300	51	38	57	54
200	51	50	55	50
100	63	97	51	58
50	59	76	43	61
10	63	74	59	73
